# Upregulation of adenylate cyclase 3 (ADCY3) increases the tumorigenic potential of cells by activating the CREB pathway

**DOI:** 10.18632/oncotarget.1324

**Published:** 2013-09-30

**Authors:** Seung-Hyun Hong, Sung-Ho Goh, Sang Jin Lee, Jung-Ah Hwang, Jieun Lee, Il-Ju Choi, Hyehyun Seo, Jong-Hoon Park, Hiromu Suzuki, Eiichiro Yamamoto, In-Hoo Kim, Jin Sook Jeong, Mi Ha Ju, Dong-Hee Lee, Yeon-Su Lee

**Affiliations:** ^1^ Cancer Genomics Branch, Research Institute, National Cancer Center, Republic of Korea; ^2^ New Experimental Therapeutics Branch, Research Institute, National Cancer Center, Republic of Korea; ^3^ Genitourinary Cancer Branch, Research Institute, National Cancer Center, Republic of Korea; ^4^ Center for Gastric Cancer, Research Institute, National Cancer Center, Republic of Korea; ^5^ Molecular Imaging and Therapy Branch, Research Institute, National Cancer Center, Republic of Korea; ^6^ Department of Biological Science, Sookmyung Women's University, Seoul, Republic of Korea; ^7^ Department of Molecular Biology, Sapporo Medical University, Sapporo, Japan; ^8^ Department of Pathology, Dong-A University College of Medicine, Busan, Republic of Korea; ^9^ Department of Life Science, Ewha Woman's University, Seoul, Republic of Korea

**Keywords:** gastric cancer, adenylate cyclase, tumorigenesis, cAMP/PKA/CREB pathway, promoter methylation

## Abstract

Adenylate cyclase 3 (*ADCY3*) is a widely expressed membrane-associated protein in human tissues, which catalyzes the formation of cyclic adenosine-3′,5′-monophosphate (cAMP). However, our transcriptome analysis of gastric cancer tissue samples (NCBI GEO GSE30727) revealed that *ADCY3* expression was specifically altered in cancer samples. Here we investigated the tumor-promoting effects of *ADCY3* overexpression and confirmed a significant correlation between the upregulation of *ADCY3* and Lauren's intestinal-type gastric cancers. *ADCY3* overexpression increased cell migration, invasion, proliferation, and clonogenicity in HEK293 cells; conversely, silencing *ADCY3* expression in SNU-216 cells reduced these phenotypes. Interestingly, *ADCY3* overexpression increased both the mRNA level and activity of matrix metalloproteinase 2 (*MMP2*) and *MMP9* by increasing the levels of cAMP and phosphorylated cAMP-responsive element-binding protein (CREB). Consistent with these findings, treatment with a protein kinase A (PKA) inhibitor decreased *MMP2* and *MMP9* expression levels in *ADCY3*-overexpressing cells. Knockdown of *ADCY3* expression by stable shRNA in human gastric cancer cells suppressed tumor growth in a tumor xenograft model. Thus, *ADCY3* overexpression may exert its tumor-promoting effects via the cAMP/PKA/CREB pathway. Additionally, bisulfite sequencing of the *ADCY3* promoter region revealed that gene expression was reduced by hypermethylation of CpG sites, and increased by 5-Aza-2′-deoxycytidine (5-Aza-dC)-induced demethylation. Our study is the first to report an association of *ADCY3* with gastric cancer as well as its tumorigenic potentials. In addition, we demonstrate that the expression of *ADCY3* is regulated through an epigenetic mechanism. Further study on the mechanism of *ADCY3* in tumorigenesis will provide the basis as a new molecular target of gastric cancer.

## INTRODUCTION

Gastric cancer is the fourth most prevalent cancer type and the second largest contributor to cancer-related deaths worldwide. In developing countries, the incidence and mortality for gastric cancer are greater (2.58 and 3.06 times higher, respectively) than those in Western countries [1]. In Korea and Japan, high incidence rates of gastric cancer have prompted the initiation of national cancer screening programs [2]. These programs have led to the diagnosis and treatment of 50% more patients with early gastric cancer than was previously possible [3,4], thereby contributing to decreased mortality [5]. However, many patients are not diagnosed until advanced stages, when metastasis to the lymph nodes or other organs has occurred [6], often rendering curative treatment impossible. Therefore, early diagnosis of gastric cancer remains an important issue, and the development of diagnostic tools such as molecular markers of gastric cancer is necessary to allow tumors to be distinguished from adjacent normal tissue.

Although a number of genes and pathways associated with gastric cancer have been identified by microarray analysis [7,8], the molecular mechanisms underlying progression of the disease are not well understood. The present study was designed to identify novel genes involved in the development of early stage gastric cancer and to shed light on their roles in tumorigenesis. We performed microarray analyses to compare gene expression profiles of human gastric cancer samples and adjacent normal tissue (NCBI GEO accession no.: GSE30727) [9]. *ADCY3* was one of the genes that were significantly overexpressed in gastric cancer samples, relative to the normal tissue.

Adenylate cyclases catalyze the formation of cyclic adenosine-3′,5′-monophosphate (cAMP), the universal second messenger, from adenosine triphosphate (ATP). The adenylate cyclase family consists of 10 members, which show high primary sequence similarity at the catalytic site [10]. Gene regulation patterns and tissue distribution appear to be family member-specific, indicating these distinct functions are important in achieving precise regulation of cellular signals in a spatiotemporal manner. Consistent with this idea, knockout and transgenic mice models have revealed distinctive physiological roles for each adenylate cyclase family member [11]. The *ADCY3* gene is located on chromosome 2p23.3 and contains 21 exons; the 4.41-kb mRNA transcript is translated into a 129-kDa protein. The *ADCY3* gene was initially identified in olfactory neuroepithelium [12] and in human islet cells [13]. *ADCY3*-knockout mice showed peripheral and behavioral anosmia [14], but to date, there has been no evidence linking *ADCY3* to tumorigenesis.

In this study, we provide the first evidence that upregulation of *ADCY3* in gastric cancer cells and tissues is associated with increased tumorigenic potential. We show that *ADCY3* upregulation increases cell migration, invasiveness, proliferation, and clonogenicity, via the activation of CREB and its downstream pathways. In addition, DNA methylation analysis of the promoter region indicates that *ADCY3* expression is regulated through an epigenetic mechanism.

## RESULTS

### Gastric cancer-specific overexpression of *ADCY3*

**Figure 1:**
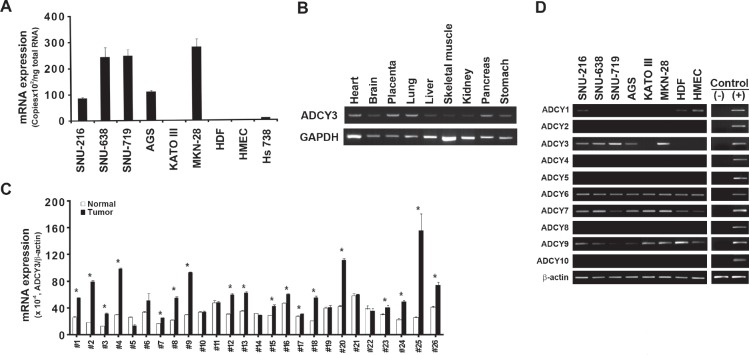
*ADCY3* mRNA expression levels in gastric cancer cells and tissues (A) Absolute quantification of *ADCY3* mRNA in gastric cancer cells (SNU-216, SNU-638, SNU-719, AGS, KATOIII, MKN28) and normal human cells (HDF, HMEC, Hs738). (B) *ADCY3* mRNA expression in various human tissues. Nine tissue samples, including heart, brain, placenta, lung, liver, skeletal muscle, kidney, pancreas, and stomach samples (Multiple-tissue cDNA panel, Clontech) were amplified. *ADCY3* expression differed in various tissues. (C) Relative quantification of *ADCY3* mRNA in gastric cancer tissues and adjacent normal tissues of Korean (#1-#21) and Japanese patients (#22-#26), * indicates *P* < 0.05 between gastric cancer vs. adjacent normal tissues. Blank and solid columns indicate normal and cancer tissues, respectively. Error bars indicate standard deviations. (D) Expression specificity of ADCY family members detected using RT-PCR in 6 gastric cancer cell lines (SNU-216, SNU-638, SNU-719, AGS, KATOIII, and MKN28) and 2 normal cells (HDF and HMEC). Among the 10 family members of *ADCY*s, *ADCY6* appeared in all cell lines tested in this analysis. *ADCY1*, *ADCY3*, and *ADCY7* showed cell line–dependent expression; *ADCY3* showed expression only in gastric cancer cell lines, but not in normal cell lines. (−) indicates the non-template negative control. (+) indicates the positive controls. The U2OS cell line cDNA was used for *ADCY1, 2, 4, 5, 10*, and U87 cell line cDNA was used for *ADCY 3, 6, 7, 8*, and *9*. Beta-actin was used as a loading control.

Comparison of gene expression profiles from microarray data revealed that *ADCY3* was significantly upregulated in gastric cancer tissues (*P* = 4.215 × 10^−10^; Table 1). We validated this by using quantitative RT-PCR to measure *ADCY3* expression levels in gastric cancer cells and tissues. *ADCY3* mRNA levels in six human gastric cancer cell lines (SNU-216, SNU-638, SNU-719, AGS, KATO III, and MKN28) were significantly higher than those in normal cell lines (HDF, HMEC, and Hs738; Figure 1A). Tissue-specific *ADCY3* expression at the mRNA level was examined in nine normal human tissues by RT-PCR. Heart, placenta, lung, and pancreas tissues showed high levels of *ADCY3* expression, whereas brain, liver, and stomach tissues showed much lower levels of expression (Figure 1B). Such organ-specific expression may indicate distinct roles for *ADCY3* in different tissues. Gastric cancer-specific overexpression of *ADCY3* was confirmed in 14 of 21 (66.7%) Korean gastric cancer tissues showing significantly higher *ADCY3* mRNA levels than those in adjacent normal tissues. Significantly elevated *ADCY3* expression was also observed in 80% of the Japanese gastric cancer cases analyzed (Figure 1C).

**Table 1:** The top 10 genes show a signi Scant differential gene expression in micro array analysis using Benjamini and Hochberg False Disco very Rate (FDR).

Gene symbol	Gene annotation	Chromosome	Position start No.	*P*-value
*MSRI*	macrophage scavenger receptor 1	8	15944161	7.530 × 10 ^−12^
*LOC44230*	similar to Interferon-induced transmembrane protein 3 (Interferon- inducible protein 1-8U)	11	305628	2.600 × 10 ^−10^
*CTSLI*	cathepsin L l	9	89530264	2.851 × 10^−10^
*IL8*	interleukin 8	4	74825107	2.691 × 10^−10^
*SERPINHI*	serpin peptidase inhibitor dade H (heat shock protein 47) member 1 (collagen binding protein 1)	11	74938382	2.975 × l0^−10^
*PTGS2*	prostaglandin-endoperoxide synthase 2 (prostaglandin G/H synthase and cyclooxygenase)	1	184907556	4.964 × 10^−10^
*LY6E*	lymphocyte antigen 6 complex locus E	8	144148702	4.281 × 10^−10^
*ADCY3*	adenylate cydase 3	2	24895545	4.215 × 10^−10^
*TULP3*	tubby like protein 3	12	2938471	4.981 × 10^−10^
*HTRAI*	HtrA serine peptidase 1	10	124211033	4.485 × 10^−10^

Because adenylate cyclase family members share significant sequence similarity, we examined their expression patterns in gastric cancer cell lines. When the gastric cancer cell lines (SNU-216, SNU-638, SNU-719, AGS, KATO III, and MKN28) and the normal cell lines (HDF and HMEC) were subjected to RT-PCR using primers specific for each family member (Figure 1D), only *ADCY3* showed a gastric cancer-specific expression pattern. *ADCY1* expression was detected in both the normal cell lines, but in only one gastric cancer cell line (SNU-216). Expression of *ADCY2*, *ADCY4*, *ADCY5*, *ADCY8*, and *ADCY10* was not detected in any of the cell lines, whereas expression of *ADCY6* was consistently detected. Different levels of *ADCY7* and *ADCY9* were detected in gastric cancer and normal cell lines. These findings suggest that *ADCY3* confers a tumorigenic effect on gastric cells, whereas the other members of the adenylate cyclase family do not.

### Clinicopathological properties of *ADCY3* in gastric cancer

**Figure 2:**
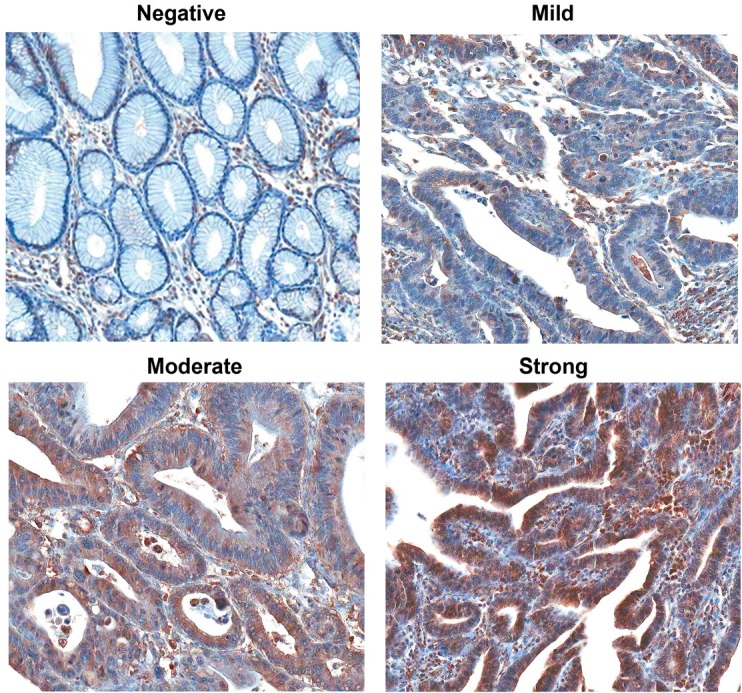
Immunohistochemical detection of *ADCY3* expression in normal and non-neoplastic gastric mucosal glands and gastric carcinomas (magnification 200x) Non-neoplastic foveolar epithelial cells did not show immunostaining. Gastric adenocarcinomas represent *ADCY3* expression with mild, moderate or strong immunostaining. Subcellular location of *ADCY3* expression was mostly cytoplasmic.

Immunohistochemical analysis showed that *ADCY3* was not expressed in non-neoplastic gastric surface and foveolar epithelial cells (Figure 2). Immunohistochemical analysis of 203 patient samples revealed 126 (62.1%) cases with a level of *ADCY3* protein expression that was consistent with the mRNA level measured by qRT-PCR. When we categorized *ADCY3* signal intensities into three levels (mild, moderate, or strong), we observed a significant association of higher *ADCY3* expression levels with the differentiated histologic subtype (*P* < 0.0001; Table 2A), as well as with Lauren's intestinal type (*P* < 0.0001, Table 2B). There was no significant association between *ADCY3* overexpression and other clinicopathological parameters, including, age, sex, depth of invasion, vascular invasion, or TNM staging.

Table 2Correlation between *ADCY3* expression and differentiation of gastric carcinomas.

**Table:** **A.** Correlation of *ADCY3* expression to differentiation grade of tubular adenocarcinoma and signet ring cell carcinoma

	ADCY3 Expression				
Histologic type	Negative (%)	Positive (%)			N
		Mild (%)	Moderate (%)	Strong (%)	
Tubular adenocarcinoma, well differentiated	8 (17.0)	9(19.2)	25 (53.2)	5 (10.6)	47
Tubular adenocarcinoma, moderately differentiated	12 (21.4)	29(51.8)	12(21.4)	3(5.4)	56
Tubular adenocarcinoma, poorly differentiated	44 (53.6)	24(29.3)	11 (13.4)	3(3.7)	82
Signet ring cell carcinoma	13 (72.2)	5(27.8)	0(0)	0(0)	18
N	77	67	48	11	203

X^2^ - test 58.742; DF:9; Significance level:*p*<0.0001; Contingency coefficient 0.474

**Table:** **B.** Correlation of *ADCY3* expression to subtypes of gastric carcinoma by Lauren's classification

	ADCY3 Expression				
Lauren classification	Negative (%)	Positive (%)			N
		Mild (%)	Moderate (%)	Strong (%)	
Intestinal	17(18.7)	31 (34.1)	36 (39.5)	7(7.7)	91
Mixed	14(45.2)	13(41.9)	3(9.7)	1(3.2)	31
Diffuse	46(56.8)	23 (28.4)	9(11.1)	3(3.7)	81
N	77	67	48	11	203

X^2^-test 37.484; DF: 6; Significance level: *p*<0.0001; Contingency coefficient: 0.395

### Role of *ADCY3* in gastric cancer tumorigenesis

**Figure 3:**
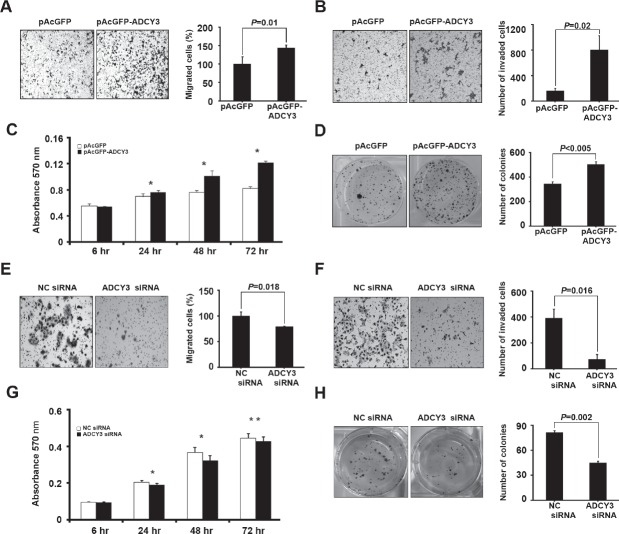
Effect of *ADCY3* expression on tumorigenesis-related cell behaviors Increment of migration, invasiveness, and colony formation in *ADCY3* overexpressing HEK293 cells (A–D). (A) Cell migration determined using a Boyden transwell chamber. Cell migration was increased by *ADCY3* overexpression. (B) Cell invasiveness was assayed in a Matrigel-coated transwell chamber. The number of cells crossing the Matrigel-coated filter was increased by *ADCY3* overexpression. (C) Cell growth of control vector cells and *ADCY3* overexpressing cells were examined using an MTT assay. * indicates *P* < 0.0001 between *ADCY3* expressing plasmid vs. control vector (D) Clonogenicity of the HEK293 cells transfected with pAcGFP-*ADCY3* showed an increase. Determination of the effects of *ADCY3* silencing on cell migration, invasiveness, and colony formation by siRNA treatment on SNU-216 gastric cancer cell line (E–H). Cell migration (E), invasiveness (F), MTT assay (G), and clonogenicity (H) of SNU-216 cells following *ADCY3*-specific siRNA treatment was decreased compared with that by negative control (NC) siRNA.

To examine the function of *ADCY3* in gastric cancer development, we investigated the effects of *ADCY3* overexpression or downregulation on tumorigenesis-related cellular characteristics such as cell migration, invasiveness, cell viability, and clonogenicity. For HEK293 cells transfected with pAcGFP-*ADCY3* (Figure S1A), the number of migrating cells significantly increased by approximately 43% (*P* = 0.01; Figure 3A). Consistent with this finding, *ADCY3*-overexpressing cells showed a 4.95-fold increase in invasiveness (*P* = 0.02) compared to cells transfected with the control vector (Figure 3B). Moreover, in MTT proliferation assays, pAcGFP-*ADCY3*-transfected cells showed higher viability than did the control cells (*P* < 0.001; Figure 3C). Clonogenic assays also revealed a reinforcement effect on anchorage-dependent cell growth (*P* < 0.005) in pAcGFP-*ADCY3*-overexpressing cells (Figure 3D). In contrast, SNU-216 gastric cancer cells treated with *ADCY3*-specific siRNA showed a loss of tumorigenesis-related cellular characteristics. *ADCY3* downregulation caused by siRNA (Figure S1B) decreased cell migration by 21% (*P* = 0.018; Figure 3E), cell invasiveness by 5.29-fold (*P* = 0.016; Figure 3F), cell viability by 12% (*P* < 0.01) following a 48-hr siRNA treatment (Figure 3G), and clonogenicity by 1.8-fold (*P* = 0.002; Figure 3H). These results collectively indicate that *ADCY3* functions during tumorigenesis of gastric cancer by changing cellular characteristics toward cancer cells.

### *ADCY3* overexpression activates CREB and upregulates *MMP2* and *MMP9*

**Figure 4:**
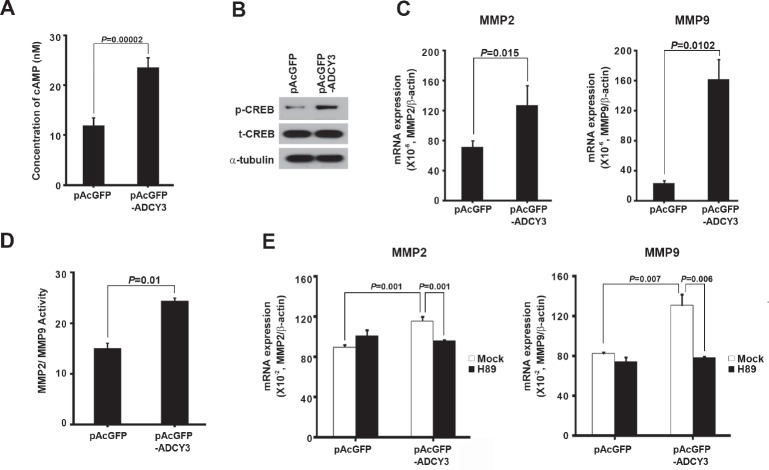
The molecular mechanism of *ADCY3* in gastric cancer tumorigenesis (A) Determination of cAMP concentration increment following forced *ADCY3* expression in HEK293 cells. The level of cAMP of ADCY3 overexpressing cells showed a twofold increase compared with the blank vector control. (B) Levels of phosphorylated CREB (p-CREB) at Ser133 were increased following *ADCY3* overexpression compared to the consistent level of total-CREB (t-CREB) between blank vector control and pAcGFP-*ADCY3* overexpression. Alpha-tubulin was used as a loading control. The mRNA expression level (C) and activities (D) of *MMP2* and *MMP9* measured in pAcGFP-*ADCY3*–transfected cells were significantly increased compared to the blank vector control. (E) After PKA inhibitor treatment, mRNA expression of *MMP2* and *MMP9* was decreased in *ADCY3* overexpressing cells.

To investigate the molecular pathway of *ADCY3* in gastric cancer tumorigenesis, we analyzed the functional consequences of *ADCY3* overexpression. First, we measured cAMP levels in pAcGFP-*ADCY3*-transfected HEK293 cells, because *ADCY3* is known to catalyze cAMP formation. In cells overexpressing *ADCY3*, we observed a 1.99-fold increase in cAMP concentration (*P* = 0.00002) compared to the control, indicating that *ADCY3* overexpression affects cAMP formation (Figure 4A). We further investigated the effects of cAMP accumulation on the progression of gastric cancer by measuring CREB activity by comparing the amounts of total CREB (t-CREB) and phosphorylated CREB (p-CREB) at the Ser133 residue. Consistent with the changes in cAMP levels that we observed, the levels of p-CREB was significantly higher in pAcGFP-*ADCY3*-transfected cells than that in control cells, whereas the level of t-CREB remained unchanged (Figure 4B). To further understand the effect of increased p-CREB on downstream pathways, we investigated the effect of changes in *ADCY3* levels on *MMP2* and *MMP9* expression and activity. *MMP2* and *MMP9* are activated by CREB and appear to play important roles in tumor invasion and metastasis [15,16]. In pAcGFP-*ADCY3*-transfected cells, in which cAMP and p-CREB were elevated, the mRNA levels of *MMP2* and *MMP9* were also significantly increased (Figure 4C). We confirmed these results by measuring the activity of *MMP2* and *MMP9* in *ADCY3*-overexpressing cells; as expected, significantly increased *MMP2/MMP9* activity was observed (*P* = 0.01; Figure 4D). Next, we examined whether the observed increase in *MMP2* and *MMP9* mRNA levels was specifically due to cAMP/PKA/CREB-mediated pathway activation. We therefore treated *ADCY3*-transfected cells with a PKA inhibitor (H89) and found that it decreased *MMP2* and *MMP9* expression levels (*P* = 0.001 and *P* = 0.006, respectively; Figure 4E). These results suggest that *ADCY3* promotes gastric cancer development via activation of cAMP-mediated CREB signaling, and upregulation of *MMP2* and *MMP9* expression.

### *ADCY3* knockdown suppresses tumor growth and cell proliferation

**Figure 5:**
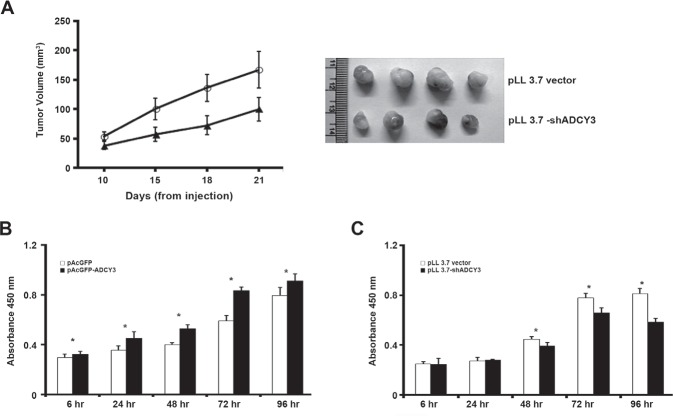
Suppression of tumor growth and cell proliferation (A) pLL 3.7 vector or pLL 3.7-sh*ADCY3* transfectants were injected subcutaneously into nude mice. Tumor growth curve (left panel) and tumor images (right panel). Cell proliferation was assessed using the BrdU incorporation assay in *ADCY3* overexpressing HEK293 cells (B) and *ADCY3* knockdown MKN28 cells (C). Cells were incubated with BrdU for 4 hr, and labeled cells were measured at absorbance 450 nm. The first absorbance measurement was performed at 6 hrs after cell plating for firm cell attachment. Further measurements were done at every 24 hrs after cell plating until 72 hrs.

To investigate the role of *ADCY3* in tumorigenesis, we used the tumor xenograft model by implanting established gastric cancer cells (MKN28) with or without *ADCY3* knockdown. We confirmed that the level of ADCY3 protein was highly decreased in *ADCY3*-knockdown MKN28 cells before implantation (Figure S1C). Nude mice were subcutaneously injected with *ADCY3*-knockdown or pLL3.7 control cells, and tumor volumes were measured three weeks after injection. Tumor volumes in mice injected with *ADCY3*-knockdown MKN28 cells were significantly lower than those in mice injected with control pLL3.7-transfected cells (Figure 5A). This result suggests that stable knockdown of *ADCY3* in gastric cancer cells can suppress tumor growth.

Next, to examine the effect of *ADCY3* expression on cell growth, we used a bromo-deoxyuridine (BrdU) incorporation assay to measure cell proliferation. The proliferation of *ADCY3*-overexpressing cells was significantly higher than that of control cells (*P* < 0.01; Figure 5B). Conversely, the proliferation of *ADCY3*-knockdown cells was significantly lower than that of control cells (*P* < 0.01; Figure 5C).

### Epigenetic regulation of *ADCY3* expression in cancer cells

**Figure 6:**
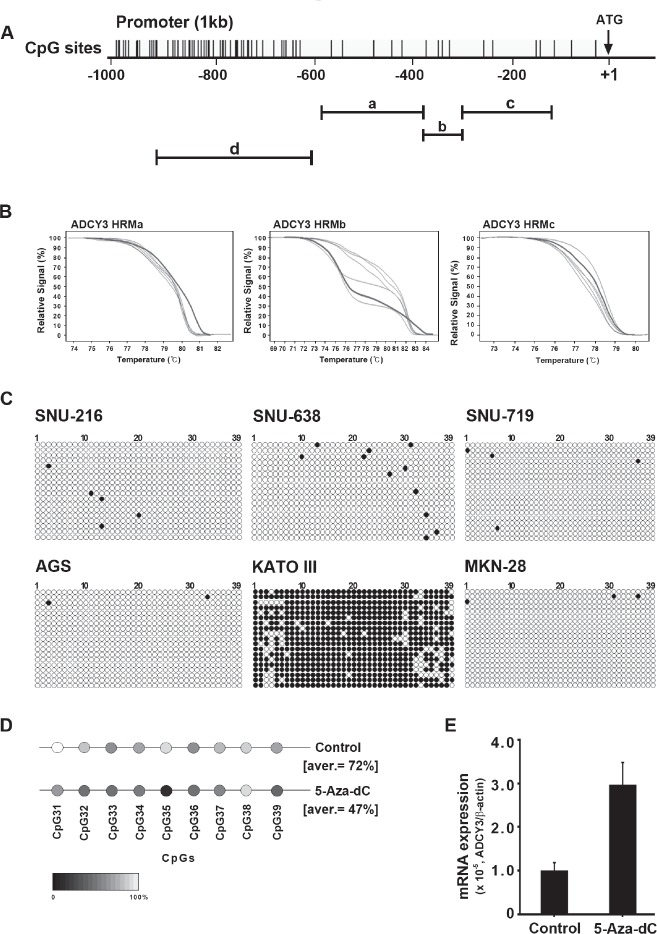
Regulation of *ADCY3* mRNA expression by DNA methylation (A) DNA methylation status of *ADCY3* in gastric cancer cell lines analyzed by bisulfite sequencing. *Top*, map of the CpG island. Individual CpG dinucleotides are indicated as vertical lines. (a, b, and c; region of MS-HRM screening, d; region of bisulfite sequencing) *Bottom*, each circle indicates a CpG site in the promoter sequence, and horizontal lines indicate the sequence of the individual clone. White and black circles depict unmethylated and methylated CpG sites, respectively. (B) Results of methylation-sensitive high-resolution melting (MS-HRM) analysis. These regions did not show significant differences in methylation status between cancer and normal cell lines. (C) Results of bisulfite sequencing. A total of 6 cell lines were surveyed for their methylation status by bisulfite sequencing, and only the KATO-III cell line showed significant methylation on region d of panel A. The demethylation by 5-Aza-dC treatment reduced *ADCY3* promoter methylation compared with negative control (D) and also restored ADCY3 expression in the KATOIII cell line according to qRT-PCR results. Control represents no 5-Aza-dC treatment (E).

The role of DNA methylation in the regulation of cancer-related gene expression has been well examined. We therefore investigated whether *ADCY3* expression is regulated by CpG methylation in the promoter region. To screen for CpG islands in the *ADCY3* promoter, we performed methylation-sensitive high-resolution melting (MS-HRM) analysis on a 600-bp region immediately upstream of the translational start site (ATG codon). We designed three sets of primer pairs to amplify PCR products spanning the 457-bp region from 585 to 129 bp upstream of the translational start site (Figure 6A). However, we did not observe clear differences in methylation levels when six different gastric cancer cell lines were analyzed (Figure 6B). MS-HRM primers could not be designed for the region 694–387 bp upstream of the translational start site, where a 308-bp CpG island was located, due to the high density of CpG sites. Instead, this region was analyzed by cloning and sequencing of bisulfite-treated DNA. We observed hypomethylation in cell lines such as SNU-216, SNU-638, SNU-719, AGS, and MKN28, in which *ADCY3* is highly expressed. Conversely, hypermethylation was observed in the *ADCY3*-non-expressing KATO III cell line (Figure 6C). Furthermore, treatment of the KATO III cell line with 5-Aza-2′-deoxycytidine (5-Aza-dC), a demethylating agent, resulted in demethylation of CpG sites in the *ADCY3* promoter (Figure 6D) and upregulation of *ADCY3* expression (Figure 6E). Taken together, these results suggest that DNA methylation of CpG islands in the promoter region indeed regulates *ADCY3* expression.

## DISCUSSION

Gastric cancer is a complex disease caused by the interaction of diverse factors [17]. Although novel genetic variations associated with gastric cancer have recently been identified using genome-wide screening [18, 19], the detailed molecular mechanisms remain largely unknown. Couple of current studies addressed the candidates of new molecular targets for gastric cancer, including *MET* amplification [20] and a epigenetically regulated tumor-suppressor, galecin-7 [21]. However, additional studies will be necessary to completely define the genetic alterations underlying tumorigenesis in gastric cancer [22] for the development of new therapeutics. Our gene expression profiling study compared cancerous and adjacent normal tissue samples from 27 Korean gastric cancer patients, and identified *ADCY3* as a candidate gastric cancer gene. We validated gastric cancer-specific upregulation of *ADCY3* by using two different methods, qRT-PCR and Immunohistochemical analysis, in 229 gastric cancer samples from patients in three different groups. We also found evidence for a functional association between *ADCY3* and gastric cancer, through CREB pathway-dependent effects on cell migration, invasiveness, proliferation, and clonogenicity. Lastly, our data also support an epigenetic mechanism for the regulation of *ADCY3* expression.

Another adenylate cyclase family member, *ADCY2*, was recently reported to be associated with altered survival in colorectal cancer [23]. This finding suggests that tissue-specific alternation of *ADCY* family members in human tissues contributes to the onset of tissue-specific diseases such as cancer. Consistent with our finding of tissue-specific *ADCY3* expression levels (Figure 1B), a survey of gene-set enrichment databases at the Broad Institute (http://www.broadinstitute.org/GSEA) revealed a highly specific expression pattern of *ADCY3* in human tissues, notably upregulation in placenta, as well as in MOLT4 leukemic cells, HL-60 cells, and B lymphoblasts.

Deregulation of the effector molecule cAMP is known to be involved in tumorigenesis [24], and reduction in cAMP has an anti-proliferative effect in colorectal cancer cells [25]. The effects of cAMP are mainly mediated by protein kinase A (PKA) [26]. cAMP/protein kinase A (PKA) signaling, the most prominent pathway downstream of the cAMP generated by ADCYs, is the primary mechanism responsible for phosphorylation of CREB [27]. Growing evidence implicates CREB as a proto-oncogene in many types of cancer [28]. In human melanoma cells, CREB has been shown to be a mediator of tumorigenesis and metastasis [29]. CREB-binding proteins are also involved in the development of Ewing sarcoma [30]. In addition, as reported for breast cancer [31], cAMP/PKA signaling regulates actin-based cell migration through cytoskeletal remodeling. Our findings suggest that the elevated cAMP levels in gastric cancer cells generated by *ADCY3* leads to increased tumor aggressiveness, as indicated by changes in properties such as cell migration, proliferation, and invasion. In our study, *ADCY3* overexpression caused a considerable increase in the mobility, invasiveness, proliferation, and clonogenicity of cancer cells, whereas silencing of *ADCY3* suppressed these activities. It has been reported that upregulation of phosphorylated CREB plays a role in the development of several cancers [27]. In particular, a correlation between phosphorylated CREB levels and tumor grading has been observed in pituitary adenoma [32], as well as in lung and prostate cancers [33]. Additionally, CREB is known to play essential roles in regulating *MMP2* and *MMP9* expression in melanoma [29] and pancreatic cancer cells [34]. The fact that the *MMP2* promoter contains a cAMP response element (CRE), at position -374 to -366 with respect to the transcription initiation site [35], supports a relationship between *ADCY3* overexpression and enhanced tumorigenic cell behavior. In summary, our results indicate that elevated *ADCY3* expression contributes to gastric cancer progression through the cAMP/PKA/CREB pathway, by increasing both mRNA expression and *MMP2* and *MMP9* activity. These changes were blocked by PKA inhibitor treatment. Therefore, upregulated *ADCY3* may lead to increased cell migration, invasion, and proliferation, which are characteristic of cancer. We used knockdown experiments to confirm the tumor-promoting effects of *ADCY3* overexpression. *ADCY3* knockdown reduced cell proliferation *in vitro* and suppressed tumor growth in a xenograft tumor model *in vivo*.

In gastric cancer, the tumorigenic potential of some proto-oncogene accounts for the amplification in cancer, such as *MET* gene amplification [20]. On the other hand, expression level of many oncogenes may be attributed by promoter activity that is subjected to epigenetic regulation as galectin-7 gene [21]. *ADCY3* expression may be affected by promoter CpG methylation, a well-known epigenetic mechanism for cancer-specific changes in gene expression. According to the encyclopedia of DNA elements (ENCODE) annotation [36], the region of the *ADCY3* promoter analyzed in our study harbors binding sites for critical transcription factors, including early growth response protein 1 (Egr-1), E2F, and activating protein 2 (AP-2), which are known to promote the development of an environment conducive for tumor formation [37,38]. It is possible that environmental factors alter the methylation status of the *ADCY3* promoter region, upregulating the expression of *ADCY3* in gastric tissue. This could lead to activation of the CREB pathway, thereby promoting tumorigenesis.

This study is the first to report that *ADCY3* is involved in cancer development. We found that *ADCY3* is specifically upregulated in gastric cancer cell lines and tissues, and that overexpression of *ADCY3* promotes tumorigenesis by increasing cell migration, proliferation, and invasiveness, through its effects on the CREB pathway. Results from a xenograft model also indicate that *ADCY3* plays a crucial role in tumor growth. As more data on selective inhibitors for individual adenylate cyclase family members will be available [11], the development of *ADCY3*-based targeted therapeutic agents for gastric cancer may become possible. In summary, although further functional and clinical validation will be necessary, our results indicate that *ADCY3* expression could be used as a prognostic marker for gastric cancer.

## MATERIALS AND METHODS

### Cell culture and tissue samples

The following cell lines were used in our study: human cancer cell lines SNU-216, SNU-638, and SNU-719 (Korea Cell Line Bank, Seoul, Korea); AGS, KATO III, MKN28, H23, A549, HCT-116, SNU-81, SK-BR-3, JIMT-1, U2OS, and U87 (ATCC, Manassas, VA, USA); human dermal fibroblast (HDF; ATCC), normal human gastric (Hs783, ATCC), and HEK293 (ATCC). Cells were cultured in appropriate growth media (Corning Cellgro, Manassas, VA, USA) supplemented with 10% (v/v) fetal bovine serum and 1x penicillin-streptomycin (Invitrogen, Carlsbad, CA, USA). Human mammary epithelial cells (HMEC; Lonza, Basel, Switzerland) were cultured according to the manufacturer's instructions. Normal and gastric cancer tissue samples (Table S1) were obtained in accordance with the principles of the Declaration of Helsinki and were approved by the institutional review board of the National Cancer Center, Korea, the Akita Red Cross Hospital, and the Sapporo Medical University, Japan, for human subject studies.

### Detection of mRNA expression by RT-PCR and quantitative RT-PCR

cDNA was synthesized from 2 μg of random primed total RNA from cultured cells using SuperScript III First-Strand Synthesis kit (Invitrogen). We obtained human cDNA from the Human Multiple Tissue cDNA panel I and Human Digestive System MTC panel (Clontech, Mountain View, CA, USA). Primers for RT-PCR or qRT-PCR were designed using Primer3 software (http://frodo.wi.mit.edu/primer3/) to span 2 consecutive exons of each gene (Table S2A). qRT-PCR reactions were performed using a LightCycler 480 (Roche Applied Science, Mannheim, Germany); results were quantified either by using β-actin for normalization (relative quantification), or by standard curve-based absolute quantification.

### Immunohistochemistry of gastric carcinoma tissues

Immunohistochemistry (IHC) of *ADCY3* was performed on 3 core cancer tissues (2 mm in diameter), including non-neoplastic gastric mucosal tissue from each patient. These were arranged as a tissue array of 4- to 6-μm sections mounted on silicon-coated glass slides. For detection, we used an anti-*ADCY3* antibody (sc-588; Santa Cruz Biotechnology, Santa Cruz, CA, USA) with the BenchMark XT automated system (Ventana Medical System, Tucson, AZ, USA), as described previously [39]. Samples were scored as negative or positive based on the staining intensity and distribution of gastric carcinoma cells; positive cases were sub-categorized into mild, moderate, or strong expression groups. We screened a total of 203 patients who had undergone gastrectomy at the Dong-A University Hospital, Busan, South Korea from January 1, 2006 through December 31, 2006. Gastric carcinomas consisted of 47 well-differentiated, 56 moderately differentiated, 82 poorly differentiated, and 18 signet ring cell adenocarcinomas. Based on Lauren's classification, there were 91 intestinal type cases, 81 diffuse type cases, and 31 mixed type cases. No patients received any preoperative treatments.

### Assessment of *ADCY3*-dependent tumorigenic cell behavior and related pathways

To overexpress *ADCY3*, HEK293 cells were transfected with pAcGFP1-ADCY3, using Lipofectamine 2000 (Invitrogen), with the empty pAcGFP-C1 vector used as a negative control. To silence the *ADCY3* gene, 5 × 10^4^ SNU-216 human gastric cancer cells were transfected with human *ADCY3*-specific small interfering RNA (siRNA) (si*ADCY3*: SI00058849 5′-ATGGAGCACCAGCTTCCTCAA-3′) or negative control (NC) siRNA (Cat. No. 1027280; Qiagen, Hilden, Germany) in a 6-well culture plate by using HiPerfect (Qiagen).

Cell migration was assayed by staining cells that had migrated downward through an 8-μm pore filter insert (BD Biosciences, Franklin Lakes, NJ, USA) during an overnight incubation at 37°C. Crystal violet (1%) was used for cell staining, and A_564_ measurements were made using a VERSAmax microplate reader (Molecular Devices, Sunnyvale, CA, USA). Invasion assays were performed by counting cells stained Diff-Quick (Sysmex, Kobe, Japan) in Matrigel-coated inserts after 48 hr of incubation. Cell viability was measured using an MTT assay. Briefly, cells were seeded in 96-well plates at 1,000 cells per well and incubated for 6 hr to allow attachment, before measurements were made. Quantification of viable cells was based on absorbance measurements at 570 nm, taken over the period from 6 to 72 hr after cell seeding. Clonogenicity was assayed by seeding 1 × 10^3^ cells per well in 6-well plates and counting the surviving colonies that were stained using Diff-Quick, after a 7-day incubation.

### Molecular assays of *ADCY3* function in gastric cancer

To confirm the function of expression of *ADCY3* and its family members, we measured cAMP level increments depending on *ADCY3* expression. HEK293 cells were transfected with 400 ng of pAcGFP or pAcGFP-*ADCY3* by using Lipofectamine 2000, and seeded in 96-well plates at a density of 2 × 10^4^ cells/well. The cAMP levels in 50 μL of cell lysate were measured using the CatchPoint cyclic-AMP Fluorescent Assay Kit (Cat# R8088, Molecular Devices) according to the manufacturer's protocol.

To measure protein expression of *ADCY3*, total CREB, and p-CREB, western blotting was performed using anti-ADCY3 (ab14778; Abcam, Cambridge, UK), anti-GFP (sc-9996, Santa Cruz Biotechnology), and anti-CREB and anti-p-CREB Ser133 (#8212, Cell Signaling Technology, Danvers, MA, USA); anti-β-actin and anti-α-tubulin (Sigma-Aldrich, St. Louis, MO, USA) antibodies were also used.

To quantify *MMP2* and *MMP9* activity, pAcGFP or pAcGFP-*ADCY3* transfected cell lysate was diluted 1:3 with activation buffer, and assayed using the Gelatinase (*MMP2*/*MMP9*) activity assay kit (Millipore, Billerica, MA, USA), following the manufacturer's protocol. For experiments involving the PKA inhibitor, pAcGFP or pAcGFP-*ADCY3* transfected cells (3 × 10^5^) were treated with 20 μM PKA inhibitor H89 (Sigma Aldrich) and incubated for 1 hr at 37°C.

### Tumor xenograft model

A lentiviral vector (pLL3.7) was used for production of *ADCY3* shRNA. Tumors were established by subcutaneously injecting 5 × 10^6^ MKN28 cells, suspended in 100 μL of PBS, into the flanks of 6-week-old BALB/c nude mice (Orient Bio Inc.). Tumor growth was monitored twice a week by measuring the width and length of tumors with calipers. Tumor volume was calculated using the formula *V = (a^2^ × b)/2*, where *a* and *b* are the width and the length in mm, respectively. Each group contained 4 or 5 animals. These experiments were performed according to institutional guidelines for the care and use of laboratory animals.

### Cell proliferation assay

Cellular DNA synthesis was measured using the bromo-deoxyuridine (BrdU) assay kit (Cell Signaling Technology, USA) according to the manufacturer's instructions. Briefly, cells were plated in 96-well plates at a density of 3 × 10^3^ cells/well and incubated for 6 hr, to allow attachment of cells before BrdU addition. BrdU incorporation by the cells was quantified using a plate reader at absorbance 450 nm. The first absorbance measurement was taken at 6 hr after cell plating, and further measurements were taken every 24 hr after cell plating, until 96 hr.

### Analysis of promoter methylation

To assay the methylation status of the *ADCY3* promoter, genomic DNA was bisulfite-treated using the EZ DNA Methylation-Gold Kit (ZYMO Research, Irvine, CA, USA). Methylation-specific PCR primers were designed using EpiDesigner (Sequenom, San Diego, CA, USA; Table S2B). A methylation-specific high-resolution melting assay (MS-HRM) was performed using 10 ng of bisulfite-treated genomic DNA and ResoLight dye in a LightCycler 480 (Roche) as described in the manufacturer's protocol. Results were analyzed using Gene Scanning software (Roche). For bisulfite sequencing, the promoter region containing 39 CpG sites was amplified from the genomic DNA of bisulfate-treated gastric cells, cloned into the TOPO-TA vector (Invitrogen), and sequenced using the 3730xl DNA analyzer (Applied Biosystems).

To investigate the effects of promoter demethylation on *ADCY3* expression, KATO III cells were incubated for 96 hr in culture medium with or without 5-Aza-2′-deoxycytidine (5-Aza-dC; Sigma Aldrich) at a final concentration of 10 μM, and the mRNA level of *ADCY3* was measured using qRT-PCR.

### Statistical analysis

The statistical significance of differences between groups was determined using Student's *t*-test. *P-*values less than 0.05 were considered statistically significant. Statistical analyses were performed using the MedCalc software (version 11.4; Mariakerke, Belgium).

## Supplementary Figure and Tables




