# Supplementary material for: Upregulation of adenylate cyclase 3 (ADCY3) increases the tumorigenic potential of cells by activating the CREB pathway
Source: Oncotarget. 2013 Sep 30;4(10):1791–803. doi: 10.18632/oncotarget.1324 (PMC3858564; doi:10.18632/oncotarget.1324)
Supplement: Supplementary file 1 [file oncotarget-04-1791-s001.doc]

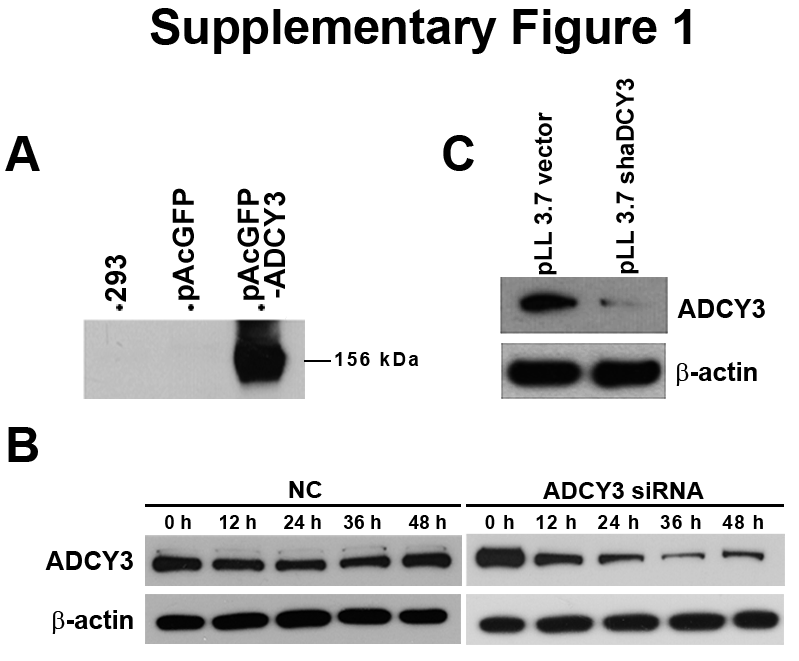


Supplementary Figure 1. Protein expression level of ADCY3 in ADCY3 overexpressing cells and silencing cells was confirmed using western blot analysis. (A) The expression level of the ADCY3 expression construct (pAcGFP-ADCY3) in HEK293 cells by transfection showed a significant increase in protein levels compared with the mock (HEK293) or control vector (pAcGFP). Protein level was detected using an anti-GFP antibody that was a designated size of the fusion protein (156 kDa) of pAcGFP (27 kDa) and ADCY3 (129 kDa). (B) ADCY3 protein expression by silencing ADCY3 mRNA expression using ADCY3-specific siRNA treatment was deceased compared with consistent levels of negative control (NC) siRNA. (C) The expression level of the ADCY3 protein decreased in ADCY3 knockdown cells compare with pLL 3.7 vector transfected cells.
