# Supplementary material for: Upregulation of adenylate cyclase 3 (ADCY3) increases the tumorigenic potential of cells by activating the CREB pathway
Source: Oncotarget. 2013 Sep 30;4(10):1791–803. doi: 10.18632/oncotarget.1324 (PMC3858564; doi:10.18632/oncotarget.1324)
Supplement: Supplementary file 2 [file oncotarget-04-1791-s002.doc]

Supplementary Table S1. Clinical information of gastric cancer patients.

**A. Korean patients for validation using qRT-PCR** (n = 21)

|  |  |  | Number of patients (%) |
| --- | --- | --- | --- |
| Ethnicity |  | Korean |  |
| Number of patients |  | Male | 14 (66.7%) |
|  |  | Female | 7 (33.3%) |
|  |  | Total | 21 |
| Age at diagnosis (years) |  | Range | 44–78 |
|  |  | Mean ± SD | 63.2 ± 10.7 |
| Disease stage | T classification | T1 | 12 (57.1%) |
|  |  | T2 | 9 (42.9%) |
|  |  |  |  |
|  | N classification | N0 | 9 (42.9%) |
|  |  | N1 | 7 (33.3%) |
|  |  | N2 | 3 (14.3%) |
|  |  | N3 | 2 (9.5%) |

**B. Japanese patients for validation using qRT-PCR** (n = 5)

|  |  |  | Number of patients (%) |
| --- | --- | --- | --- |
| Ethnicity |  | Japanese |  |
| Number of patients |  | Male | 3 (60.0%) |
|  |  | Female | 2 (40.0%) |
|  |  | Total | 5 |
| Age at diagnosis (Years) |  | Range | 56–82 |
|  |  | Mean ± SD | 68.0 ± 10.7 |
| Disease stage | T classification | T1 | 4 (80.0%) |
|  |  | T2 | 0 (0%) |
|  |  |  |  |
|  | N classification | N0 | 3 (60.0%) |
|  |  | N1 | 0 (0%) |
|  |  | N2 | 1 (20.0%) |
|  |  | N3 | 0 (0%) |

Supplementary Table S2A. Oligonucleotide primer sequences used in RT-PCR or qRT-PCR

| Primer Name | Sequence (5'→3') |
| --- | --- |
| ADCY1-F | CCATCCCCAACTTCAATGAC |
| ADCY1-R | AGGTGGGAGGAGATGGACTT |
| ADCY2-F | CCATGGTGGAGTTTGCTTTT |
| ADCY2-R | TGACAGTGTTGCCCCAGATA |
| ADCY3-F | GAGTCACCCCCGATGTCAAC |
| ADCY3-R | TTGCCCCAGATGTCGTAGTG |
| ADCY4-F | CTCTCCAAGCCCAAGTTCAG |
| ADCY4-R | ACTACGGGTCCATGGTTCAA |
| ADCY5-F | AGTGTGTGGCGGTCATGTT |
| ADCY5-R | CTGCCGATGGTCTTGATCTT |
| ADCY6-F | GAGGCAAACAATGAGGGTGT |
| ADCY6-R | TAGTCAGCCAGGGCAGTGAT |
| ADCY7-F | GGCGTGGAGAAGATCAAGAC |
| ADCY7-R | CGGAAGGAGTTGAAGGAGTG |
| ADCY8-F | TGCTGACTTCGATGAGTTGC |
| ADCY8-R | GTCAGGGCGAGTGAGAAGTC |
| ADCY9-F | GCTGCTCCTGCTCTACGTCT |
| ADCY9-R | AGGCGGTAGCTGACTTCAAA |
| ADCY10-F | TAGGTACATGGAGGGGCAAG |
| ADCY10-R | GACGTAAGCCATCAGGTGGT |
| MMP2-F | TACGATGATGACCGCAAGTG |
| MMP2-R | CTCCTGAATGCCCTTGATGT |
| MMP9-F | GGGAAGATGCTGCTGTTCA |
| MMP9-R | TCAACTCACTCCGGGAACTC |
| β-actin-F | CATCGAGCACGGCATCGTCA |
| β-actin-R | TAGCACAGCCTGGATAGCAAC |

Supplementary Table S2B. Oligonucleotide primer sequences used in analysis of promoter methylation.

| Primer Name | Sequence (5'→3') |
| --- | --- |
| ADCY3-HRMa-F | CCATCCCCAACTTCAATGAC |
| ADCY3-HRMa-R | AGGTGGGAGGAGATGGACTT |
| ADCY3-HRMb-F | CCATGGTGGAGTTTGCTTTT |
| ADCY3-HRMb-R | TGACAGTGTTGCCCCAGATA |
| ADCY3-HRMc-F | GAGTCACCCCCGATGTCAAC |
| ADCY3-HRMc-R | TTGCCCCAGATGTCGTAGTG |
| ADCY3-BS-F | CTCTCCAAGCCCAAGTTCAG |
| ADCY3-BS -R | ACTACGGGTCCATGGTTCAA |
